# An experimental insight of the hydrodynamics of microjet assisted fluidized bed of ultrafine particles

**DOI:** 10.1038/s41598-025-22982-4

**Published:** 2025-11-11

**Authors:** Syed Sadiq Ali, S. K. Safdar Hossain, M. E. Ali Mohsin, Mohammad Asif

**Affiliations:** 1 Department of Chemical Engineering, King Faisal University, PO Box 380, 31982 Al-Ahsa, Saudi Arabia; 2 Department of Chemical Engineering, King Saud University, PO Box 800, 11421 Riyadh, Saudi Arabia

**Keywords:** Fluidization, Agglomeration, Microjet, Bed expansion, Persistence, Chemical engineering, Characterization and analytical techniques, Nanoparticles

## Abstract

Strong interparticle forces (IPFs) in ultrafine powders lead to agglomeration and poor fluidization quality posing a major challenge in many industrial processes. This study investigates the use of a high-energy microjet to improve the fluidization of nanosilica. A 200 μm nozzle was used to inject a secondary flow at a sonic velocity of 312 m/s, creating intense, localized turbulence. Analysis of local pressure drop transients confirmed that this energy input effectively de-agglomerated the powder, particularly in the lower bed. The combination of the microjet with a primary inlet gas flow produced a substantial improvement in fluidization, achieving a maximum bed expansion of approximately ten times the initial height. Furthermore, by conducting successive runs, we demonstrated a significant persistence effect. The bed retained its improved hydrodynamic characteristics, responding more rapidly in subsequent runs, implying an irreversible microjet-induced change in the powder’s agglomerate structure.

## Introduction

The gas phase dispersion of powders using fluidization ensures efficient fluid–solid mixing and significantly enhances heat and mass transfer rates compared to conventional fixed-bed operations. This characteristic greatly improves surface-based rate processes, especially in catalyst-driven reactions, as well as in the production of pharmaceuticals, cosmetics, biomaterials, and related applications^1–6^.

While fluidization is generally effective for Geldart’s Group A and B powders, ultrafine powders pose a challenge due to strong inter-particle forces (IPFs) arising from their small particle size^7–11^. These IPFs cause particle aggregation, resulting in the fluidization of agglomerates rather than individual particles^12–15^. This behavior leads to poor bed expansion and high incipient fluidization velocities^16–20^. Furthermore, variations in agglomerate size induce size-based segregation along the bed height, complicating the fluidization process. The upper bed region, containing smaller agglomerates, tends to fluidize earlier than the lower region, which is dominated by larger agglomerates, resulting in height-dependent variations in fluidization hydrodynamics^18^. The agglomerate size disparity has been pointed out in bed collapse studies as well. In a related study, Ali et al.^21^ reported that the upper bed layer comprised smaller agglomerates, ranging from 10 to 30 μm, while the lower layer contained significantly larger, more rigid agglomerates, ranging from 100 to 300 μm. Similarly, another study observed agglomerate sizes reaching up to 2000 μm in the lower layer, with the upper layer containing agglomerates approximately ten times smaller^22^.

Addressing the challenges of fluidizing ultrafine powders often requires supplemental energy input. Several methods have been proposed to introduce this energy into the fluidized bed. Mechanical vibration and high-shear mixing have been explored to enhance fluidization performance^23–25^. However, these techniques can be energy-intensive and may necessitate substantial modifications to fluidized bed design, potentially limiting their practicality for industrial-scale applications. Acoustic vibration has also been investigated as an alternative^26–28^. When applied at the system’s resonance frequency, acoustic excitation has been shown to effectively promote deagglomeration and improve fluidization quality^29–31^. Another promising strategy involves pulsing the inlet flow, which has demonstrated improvements in bed homogeneity, a reduction in minimum fluidization velocity, and a decrease in agglomerate size^32–36^. Particle premixing, aimed at modifying the interparticle force equilibrium to limit agglomerate growth, is another approach^37–39^. However, it poses contamination risks and often requires post-processing separation. In some cases, combined techniques have yielded superior results. For instance, the simultaneous application of pulsed flow and particle premixing has shown enhanced bed homogeneity and greater agglomerate size reduction compared to either technique alone^40,41^.

Microjets, commonly used in spray drying, paint industries and the pharmaceutical industries for producing fine, uniform particles, have also been explored as a method to enhance powder fluidization quality^42,43^. When fluid enters the bed at sonic velocity, it generates intense turbulence, improving fluid–solid interactions. Quevedo et al.^44^ conducted a comprehensive study on microjet-assisted fluidization, systematically varying parameters such as nozzle number, flow direction, length, and placement across different powders. Bed expansion, assessed by monitoring bed height, revealed significant deagglomeration due to microjet-driven energy input, with bed expansion reaching several times the initial height. Such a substantial improvement in fluidization hydrodynamics has not been reported with other assistance techniques. In addition, they also conducted fluidization studies in a larger column of 12.7 cm diameter in order to replicate the performance in the industrial applications. They found that the microjet assistance exhibited good results; the bed expansion was up to five times the original bed height for Aeroxide TiO2 P25 particles when the microjet was directed downwards towards the distributor of the column. The upward directed microjet generated lower bed expansion than the downward directed microjet. Optimal performance was achieved with microjet nozzles positioned near the distributor and directed downward. Multiple microjets can be employed for even larger columns^45,46^. Yang et al.^47^ utilized X-ray tomography to investigate bubble dynamics in microjet-assisted fluidization of fine powders with mean diameter of 76 μm, proposed a correlation to predict bubble penetration depth. An and Andino^48^ studied microjet and vibration-assisted fluidization of TiO₂ nanoparticles, identifying optimal operational parameters for enhanced fluidization performance.

While microjet-assisted fluidization has been studied, a comprehensive understanding of both local and global bed dynamics is still lacking in the literature. This knowledge is crucial for accurately evaluating the efficacy of microjet-assisted fluidization, especially for large-scale industrial applications. A significant, yet often neglected, aspect of fluidization assistance promoted deagglomeration is the persistence effect: whether agglomerates revert to their original size after the cessation of supplemental energy input. Typically, the particles in the bed, which deagglomerated under the effect of external energy input by assistance, tend to aggregate back to their initial size range due to interparticle forces (IPFs) when external energy is removed, resulting in consistent behavior across successive runs. On the contrary, persistent deagglomeration leads to progressively particulate behavior with repeated operations, indicating a sustained disruption of agglomerate structures. To investigate this phenomenon, a precisely engineered micro-nozzle system delivering secondary flow at a sonic velocity of 312 m/s was implemented in a fluidized bed of nano-silica, which exhibits agglomerate bubbling fluidization (ABF). The ABF is the behavior exhibited by the ultrafine particles bed, whereby the particles tend to aggregate to form different sized agglomerates and the fluid flow occurs through channels and cracks. This results in poor expansion of the bed, high incipient fluidization and low fluidization quality^16^. Local pressure transients in different regions along the bed height were monitored, and successive experimental runs were conducted to assess the persistence of microjet-induced deagglomeration. The bed’s transient response was modeled to quantify the extent and durability of this persistence effect.

## Theory

Fluid flow is considered compressible when density variations exceed 10%. Therefore, for high-pressure systems, compressibility effects must be accounted for^49^. The Mach number, a crucial parameter in analyzing high-speed fluid flow, is defined as:

1 $$M = \frac{v}{c}$$

where $v$ represents the fluid velocity and $c$ the speed of sound. Flow regimes are classified based on the Mach number: sonic or critical flow occurs when $M=1$, subsonic flow when $M<1$, and supersonic flow when $M>1$. Fluid compressibility becomes significant when $M$ approaches or exceeds 0.3. For an ideal gas, the speed of sound is given by

2 $$c = \sqrt {\frac{kRT}{{M_{w} }}}$$

where $$R$$ is the universal gas constant,$$T$$ is the absolute temperature, $${M}_{w}$$ is the molecular weight and $$k = {\raise0.7ex\hbox{${C_{p} }$} \!\mathord{\left/ {\vphantom {{C_{p} } {C_{v} }}}\right.\kern-0pt} \!\lower0.7ex\hbox{${C_{v} }$}}$$ (valid for ideal gas).

**Fig. 1:**
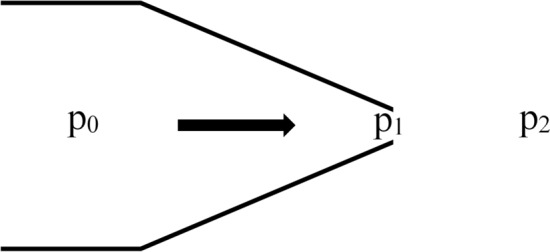
Flow through a nozzle.

Considering isentropic (reversible adiabatic) nozzle flow as shown in Fig. 1, the flow conditions at the nozzle can be given as:

3 $$\frac{{p_{0} }}{{p_{1} }} = \left( {1 + \frac{k - 1}{2}M_{1}^{2} } \right)^{{{\raise0.7ex\hbox{$k$} \!\mathord{\left/ {\vphantom {k {k - 1}}}\right.\kern-0pt} \!\lower0.7ex\hbox{${k - 1}$}}}}$$

4 $$\frac{{T_{0} }}{{T_{1} }} = 1 + \frac{k - 1}{2}M_{1}^{2}$$

5 $$\frac{{\rho_{0} }}{{\rho_{1} }} = \left( {1 + \frac{k - 1}{2}M_{1}^{2} } \right)^{{{\raise0.7ex\hbox{$1$} \!\mathord{\left/ {\vphantom {1 {k - 1}}}\right.\kern-0pt} \!\lower0.7ex\hbox{${k - 1}$}}}}$$

The mass velocity $$G = {\raise0.7ex\hbox{$w$} \!\mathord{\left/ {\vphantom {w A}}\right.\kern-0pt} \!\lower0.7ex\hbox{$A$}}$$ is the ratio of mass flow rate $$w$$ and cross-sectional area of the nozzle exit $$A$$. Using Eqs. (1)–(4), $$G$$ can be calculated as:

6 $$G = \sqrt {\frac{{kM_{w} }}{{RT_{0} }}} M_{1} \left( {1 + \frac{k - 1}{2}M_{1}^{2} } \right)^{{ - \frac{k + 1}{{2\left( {k - 1} \right)}}}}$$

For choked or sonic flow ($M$=1, denoted by the subscript ‘o’):

7 $$M_{1} = M_{1}^{^\circ } = 1$$

Hence, the above Eqs. (3)–(6) can be written as:

8 $$\frac{{p^{^\circ } }}{{p_{0} }} = \left( {\frac{2}{k + 1}} \right)^{{{\raise0.7ex\hbox{$k$} \!\mathord{\left/ {\vphantom {k {\left( {k - 1} \right)}}}\right.\kern-0pt} \!\lower0.7ex\hbox{${\left( {k - 1} \right)}$}}}}$$

9 $$\frac{{T^{^\circ } }}{{T_{0} }} = \frac{2}{k + 1}$$

10 $$\frac{{\rho^{^\circ } }}{{\rho_{0} }} = \left( {\frac{2}{k + 1}} \right)^{{{\raise0.7ex\hbox{$1$} \!\mathord{\left/ {\vphantom {1 {\left( {k - 1} \right)}}}\right.\kern-0pt} \!\lower0.7ex\hbox{${\left( {k - 1} \right)}$}}}}$$

11 $$G^{^\circ } = p_{0} \sqrt {\frac{{kM_{w} }}{{RT_{0} }}} \left( {\frac{2}{k + 1}} \right)^{{\frac{k + 1}{{2\left( {k - 1} \right)}}}}$$

In order to calculate the discharge rate from the nozzle, the critical pressure, ${p}^{^\circ }$, is calculated using Eq. (8). If ${p}_{2}>{p}^{^\circ }$, then the flow is subsonic and ${p}_{1}={p}_{2}$, and the important parameters can be calculated by using Eqs. (3) – (6). However, if ${p}^{^\circ }\ge {p}_{2}$, the flow is sonic and ${p}_{1}={p}^{^\circ }$. The mass velocity can be calculated using Eq. 11, while exit temperature and density at sonic conditions can be calculated by using Eqs. (9)–(10) respectively.

In the present study, $${p}_{0}=120 psi=827.4 kPa$$ and $${p}_{2}=1 atm=101.3 kPa$$. With $$k=1.4$$ for nitrogen gas, the critical pressure '$${p}^{^\circ }{\prime}$$ is calculated using Eq. 8 as $${p}^{^\circ }=437.1 kPa$$. Since $${p}^{o}>{p}_{2}$$, the flow is sonic. Hence, $${p}_{1}=437.1 kPa$$ and $$M=1$$. G^o^ was calculated from Eq. 11 which is, $$G^{o} = 1938.24 {\raise0.7ex\hbox{${kg}$} \!\mathord{\left/ {\vphantom {{kg} {m^{2} s}}}\right.\kern-0pt} \!\lower0.7ex\hbox{${m^{2} s}$}}$$. Therefore, the mass flow rate is $$w = 6.09 \times 10^{ - 5} {\raise0.7ex\hbox{${kg}$} \!\mathord{\left/ {\vphantom {{kg} s}}\right.\kern-0pt} \!\lower0.7ex\hbox{$s$}}$$ and the volumetric flow rate is calculated as $$\dot{V}=2.826 LPM$$. The sonic velocity at nozzle exit, calculated from Eq. (1), is $$v = 312.3 {\raise0.7ex\hbox{$m$} \!\mathord{\left/ {\vphantom {m s}}\right.\kern-0pt} \!\lower0.7ex\hbox{$s$}}$$.

## Experimental

**Fig. 2:**
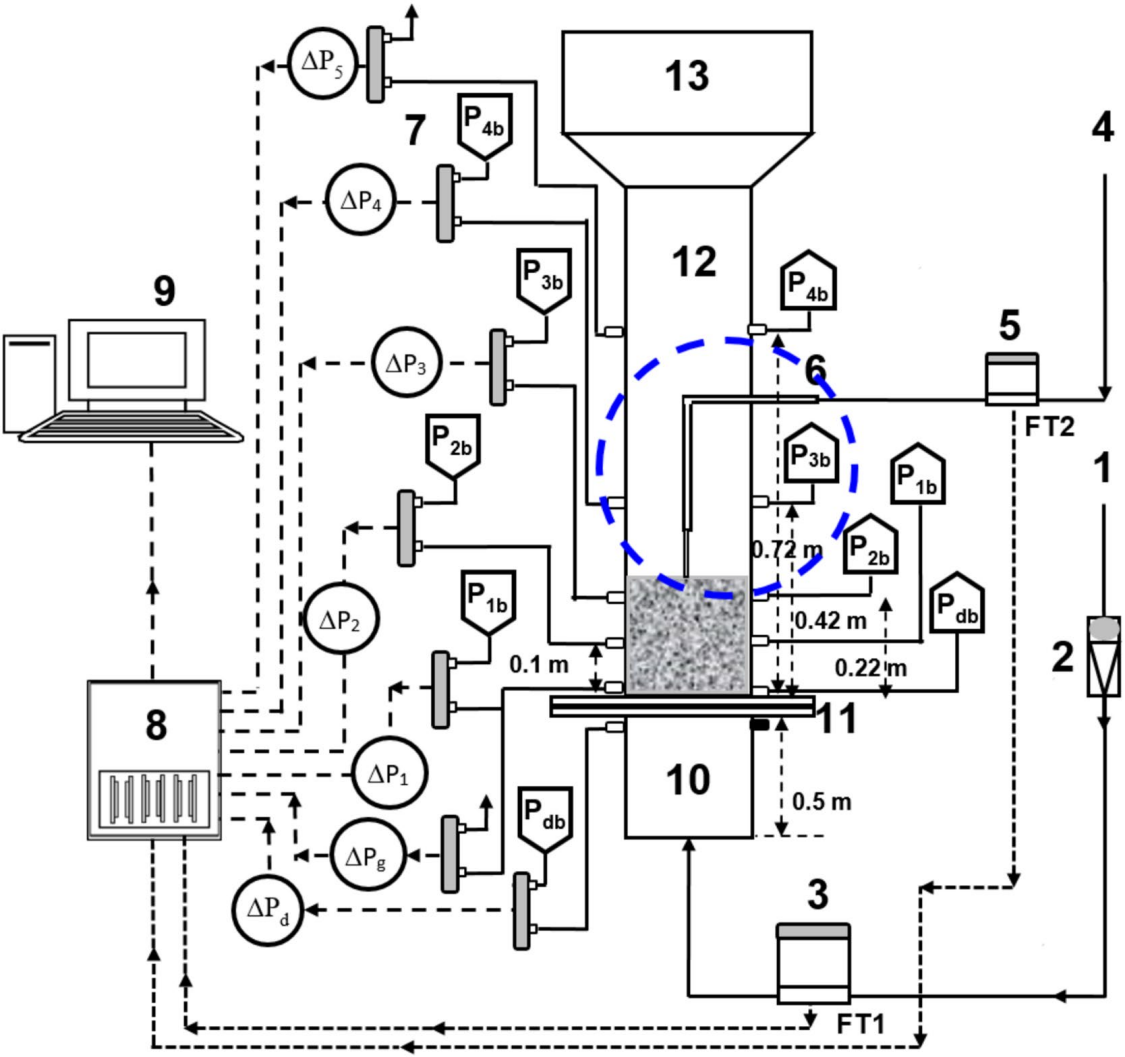
Schematic of the microjet experimental set-up: (1) Compressed air; (2) Flow meter; (3) Flow totalizer; (4) Nitrogen supply from nitrogen cylinder; (5) Flow totalizer; (6) Microjet set-up; (7) Pressure transducers; (8) Data Acquisition System Calming section; (9) Computer; (10) Calming section; (11) Distributor; (12) Test section; (13) Disengagement section.

The schematic of the experimental set-up is illustrated in Fig. 2. The primary fluid flow entered the test section through a distributor, which is a perforated disc with 2 mm holes in a circular pitch and 2.5% open area, and ensured a uniform flow throughout the cross-sectional area of the test section. A calming section of length 0.3 m was fitted before the test section to mitigate any turbulence in the fluid before entering the test section. The disengagement section of length 0.2 m and diameter 0.14 m was installed after the test section. Also, a tapered section was fitted between test section and disengagement section so that the elutriating particles can slide back in the test section. The fluid used for the primary flow is compressed ambient air. The Gilmont flow meters were used to set the initial primary fluid flow.

**Fig. 3:**
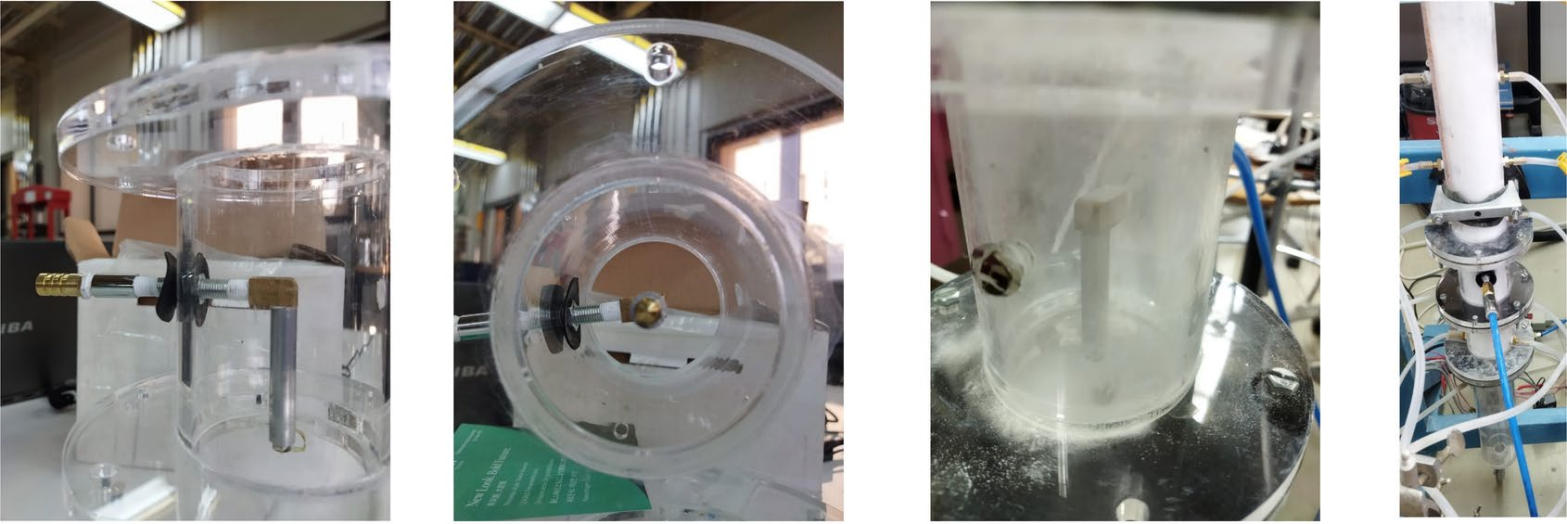
Snapshots of the microjet set-up.

The microjet set-up was fabricated using an arrangement of steel tubes and fittings of 0.6 cm ID approximately, fitted with a nozzle with inner diameter of 220 μm at the microjet exit. The microjet arrangement is encircled in blue in Fig. 2, while the snapshots of the microjet arrangement are displayed in Fig. 3. The microjet was precisely and accurately set in the center of the test section, facing vertically downwards towards the distributor. Since, the line pressure was very high at 827.4 kPa (120 psi) and the leakages are likely to happen, the fittings were very carefully tightened and regular leak tests were performed. A Bronkhorst flow totalizer (EL-FLOW Select F-201CV-20K-AAD-11-V), which can operate efficiently at high pressure, was used to measure flow rate of N_2_ through the microjet.

The pressure transients were measured as voltage signals using fast-response pressure transducers of high accuracy and a suitable range at a sampling frequency of 100 data/s. The local pressure transients were recorded at five different locations of the bed in order to obtain the detailed hydrodynamics of the bed. The location of the pressure taps are reported in Table 1. The voltage signals from the pressure transducers were recorded using a data acquisition system (DAQ) and further processed using Matlab software.

**Table 1:** Pressure tap positions to record pressure drop in different regions of test section.

Pressure drop	Bed region	Pressure tap positions (from the distributor)
${\Delta {\varvec{P}}}_{1}$	Lower	0.05 m–0.095 m
${\Delta {\varvec{P}}}_{2}$		0.095 m–0.24 m
${\Delta {\varvec{P}}}_{3}$	Lower-middle	0.24 m–0.44 m
${\Delta P}_{4}$	Middle	0.44 m–0.75 m
${\Delta P}_{5}$	Upper	0.75 m–Open
${\Delta P}_{g}$	Overall	0.05 m–Open

**Fig. 4:**
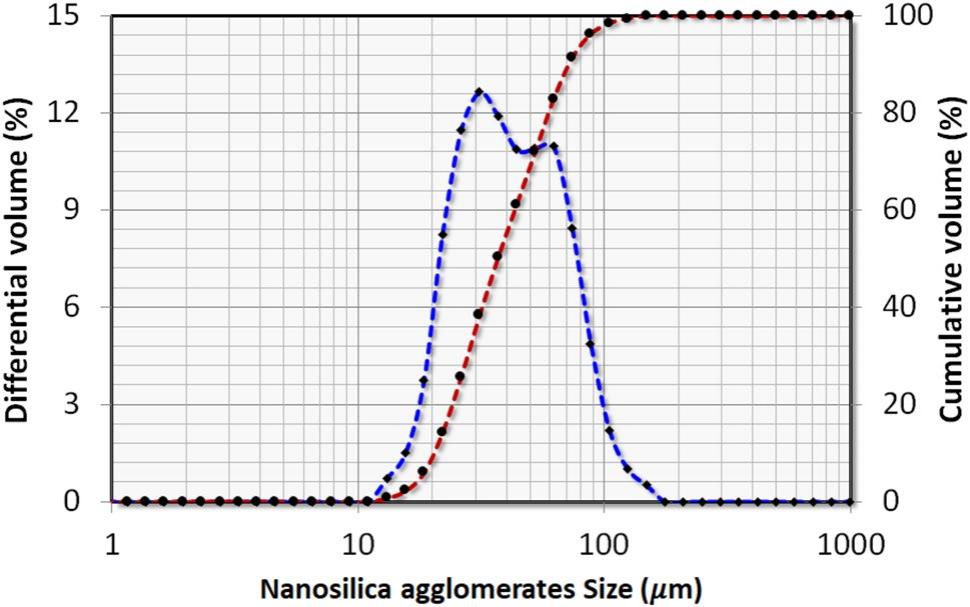
Particles size analysis of nanosilica powder.

The nanosilica particles used for the experiments is hydrophilic fumed silica, Aerosil 200 of primary size of 0.012 µm and the corresponding BET surface area is 200 ± 25 m^2^/g. However, under the effect of IPFs and cohesive forces, the nanosilica particles aggregate to develop different sized agglomerates. The particles size analysis data reported in Fig. 4 indicate that the nanosilica particles’ size range from 10 to 150 µm due to agglomeration phenomenon. In addition, the true density of the particles is 2200 kg/m^3^, but its tapped density is significantly low up to 50 kg/m^3^, which results in very high bed voidage of 0.98^33^.

Two sets of experiments were performed using nanosilica particles. The first set was performed at ‘zero primary flow rate’ and the other set of experiments were performed by varying the primary flow rate from 0.0 mm/s to 35 mm/s. The secondary (microjet) flow was set at 312 m/s and the initial static bed height was maintained at 0.16 m.

## Results and discussion

### Microjet effect at primary flow of 0.0 mm/s and microjet flow of 312 m/s

**Fig. 5:**
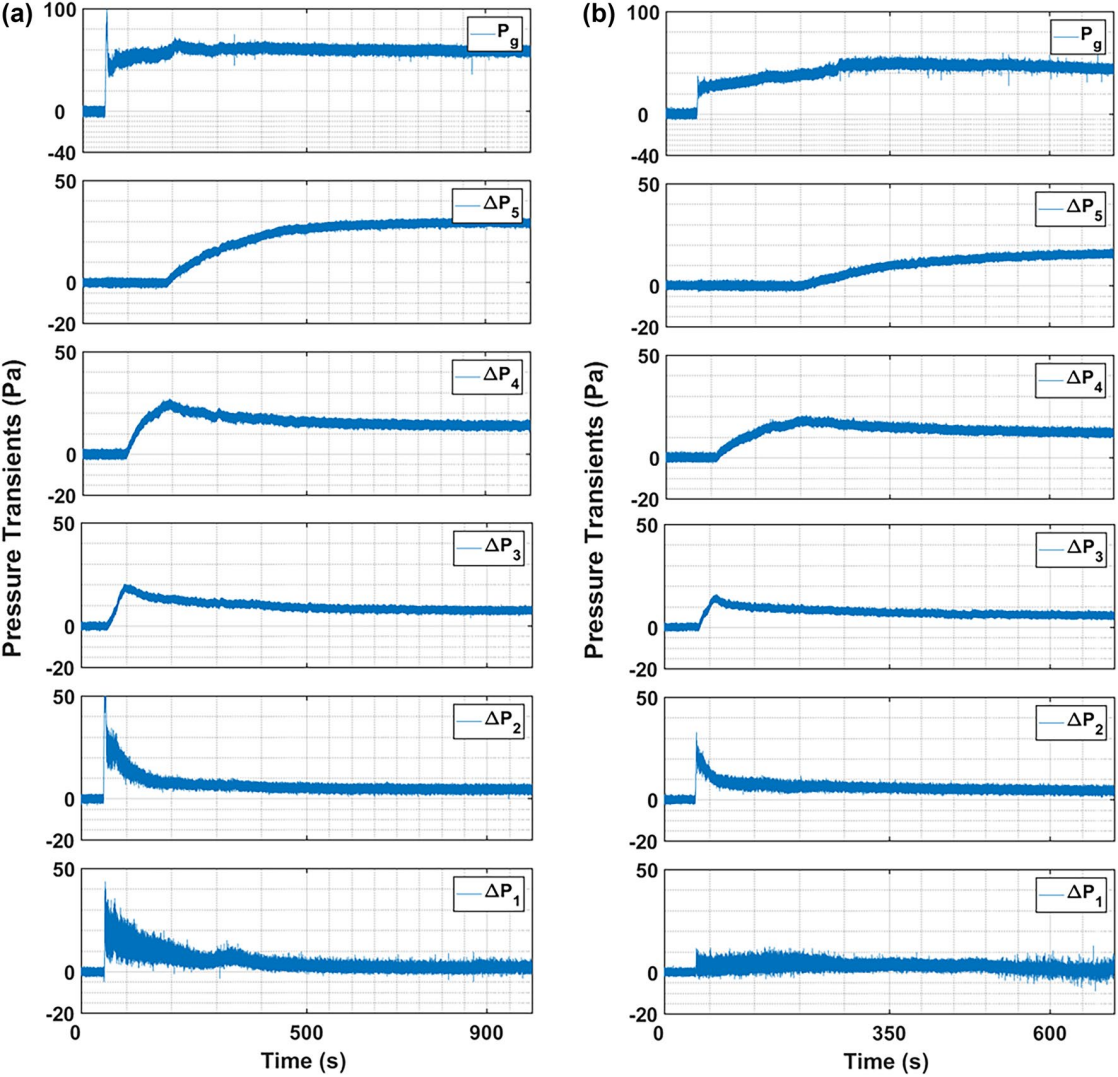

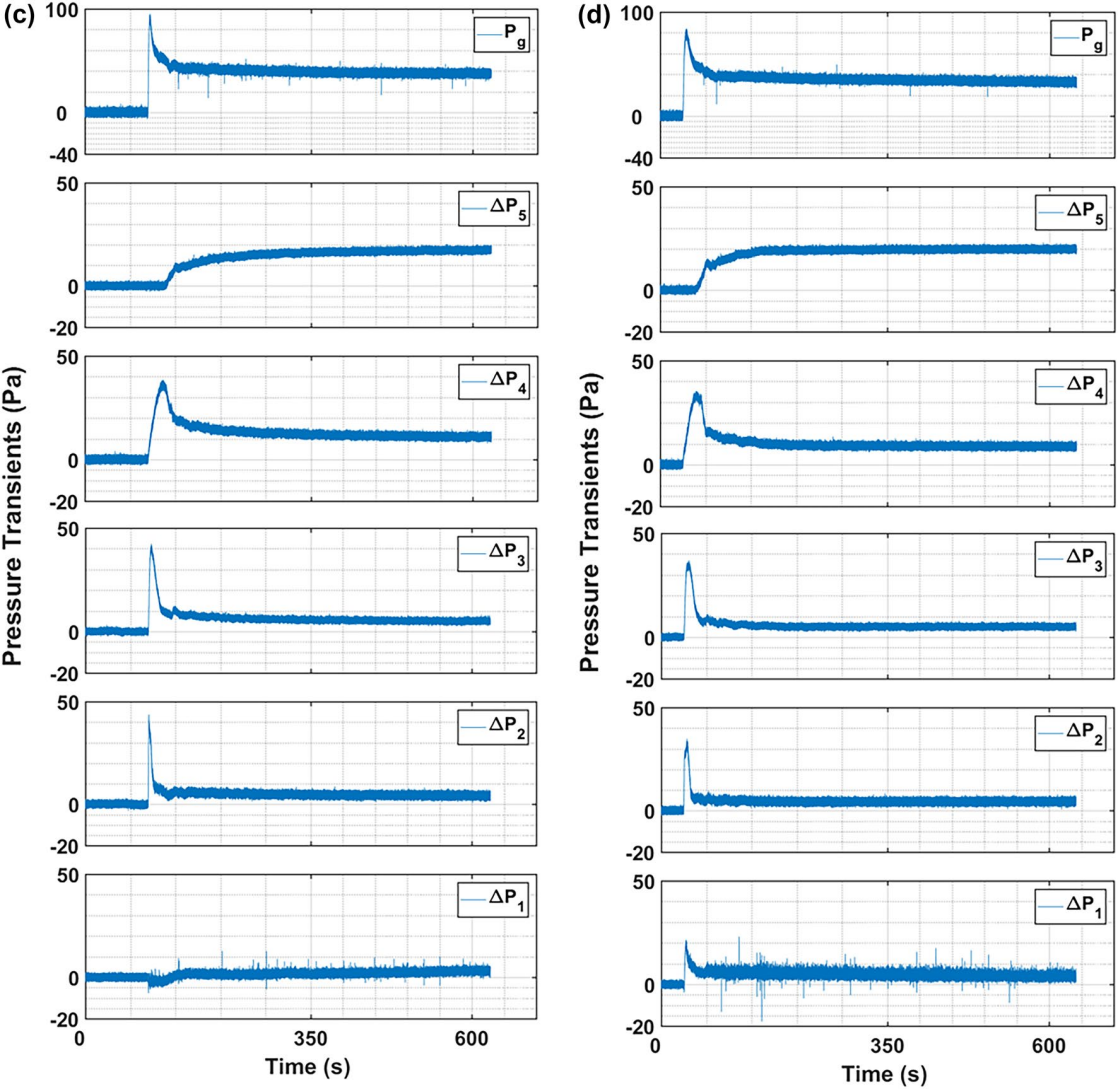
Region-wise pressure drop transients of nanosilica during the microjet flow with bed height 16 cm, when primary flow is 0 mm/s while microjet flow is 312 m/s; (**a**) Fresh powder added first time; (**b**) Run 2 performed after1 hr of first run; (**c**) Run 3 performed after 3 h of first run; (**d**) Run 4 performed after 18 h of first run.

Figure 5 reports the effect of repeated microjet flow on the nanosilica bed hydrodynamics. The nanosilica powder used in the first experiment was subjected to microjet flow in three subsequent experiments performed at different time interval. Figure 5–a illustrates the pressure transients using fresh nanosilica up to the bed height of 0.16 m. The bed height was chosen so that the bed meniscus was above the microjet nozzle. The microjet flow was begun after few seconds of initial pressure transients recording. The sudden increase in pressure transients at ${\Delta P}_{1}$ at around 48 s marks the initiation of the microjet flow. The impact of microjet flow can be noticed simultaneously in ${\Delta P}_{1}$ and ${\Delta P}_{2}$ pressure transients since the powder was present up to the ${\Delta P}_{2}$ region. A sudden increase in pressure drop can be seen followed by the vigorous pressure fluctuations. The pressure fluctuations steadily mitigated and after a period of time, the pressure drop subsided to a steady value. As visually observed as well, with the initiation of microjet flow there was instantaneous high turbulence in the bed and the particles moved vigorously displaying a chaotic motion throughout the bed, which can be correlated with the initial pressure fluctuations at ${\Delta P}_{1}$ region in Fig. 5–a. Furthermore, after the initial turbulence, the expansion in the bed was visible, whilst the intensity of turbulence steadily decreased, marked by the pressure transients’ mitigation and attainment of steady state in ${\Delta P}_{1}$ region. The similar pressure transients’ behaviour was recorded in ${\Delta P}_{2}$ region as well, but with lesser intensity. The nanosilica bed constituted of uneven sized agglomerates bound under the strong cohesion forces, which readily deagglomerated when the bed was subjected to fluid flow with intense velocity through microjet. The fine particles developed due to the initial turbulence expanded and moved to the upper bed region and eventually resulted in the rise of pressure transients consecutively in ${\Delta P}_{3}$, ${\Delta P}_{4}$ and ${\Delta P}_{5}$ region in Fig. 5–a after 5-s, 29-s and 164-s respectively. It is imperative to mention here that the nanosilica bed generally exists in multiple segregated regions based on agglomerates size, with size increasing from the bottom towards the top of the column^50^. The hard agglomerates that remained impervious to the microjet effect was left in the lower bed region, subsequently exhibiting a steady pressure drop. The complete bed attained the steady state after around 552-s and no apparent bed expansion was noticed further.

Another experimental run was conducted as ‘Run 2’ after two hours of the completion of the first run. The fresh nanosilica powder was not used in this run, but the nanosilica present in the bed after ‘Run 1’ was again subjected to microjet flow with sonic velocity. The nanosilica bed completely settled and was static in two-hour period. Generally, the ultrafine powders reagglomerate due to cohesive forces once out of fluidization environment and exhibit similar fluidization behaviour as in the first run when re-subjected to the fluidization experiment. The powder was reused to determine if the reagglomeration has reoccurred in the present instance. The pressure transients for ‘Run 2’ are reported in Fig. 5–b. The intensity of the initial turbulent pressure transients’ fluctuations in the lower bed region (${\Delta P}_{1}$ and ${\Delta P}_{2}$) have almost subdued as compared to the previous run. Also, the bed expanded swiftly, reaching ${\Delta P}_{4}$ region in almost 28 s after the commencement of microjet flow, which is approximately half of the time required in the first run. The phenomenon clearly indicates that the nanosilica bed retained the deagglomerated state attained during Run 1 and the nanosilica bed, which is homogeneous comprising of finer particles, fluidized instantaneously with the initiation of microjet flow resulting in a quick expansion of the bed.

Henceforth, the third and fourth runs were performed using the same powder in the column previously used in Run 1 and Run 2. The third run was repeated after two hours of the second run, while the fourth run was conducted after twelve hours of the third run. The pressure transients reported in Fig. 5 c–d indicates that there were no noticeable pressure transients’ fluctuations in the lower bed region and the transients have completely subdued during the initial time period of microjet flow. The bed expanded smoothly and swiftly with the commencement of microjet flow. The expanding bed reached the ${\Delta P}_{4}$ region in approximately 5-s during both runs. The bed behaviour in runs subsequent to the initial one indicates a persistent state of deagglomeration. This confirms that the microjet alters the balance of interparticle forces in the nanosilica bed to a degree that is maintained after the flow ceases. Since the nanosilica particles were fully suspended in the fluid especially in the upper bed region (${\Delta P}_{4}$ and ${\Delta P}_{5}$), the steady pressure drop attained is equivalent to the effective bed weight in the fluidized bed can be a reasonable approximation here. The expression can be used effectively to calculate the present particles’ mass in each region as:

12 $$\frac{\Delta P}{{\Delta {\text{L}}}} = \left( {{\uprho }_{{\text{b}}} - {\uprho }_{{\text{f}}} } \right){\text{g}} = { }\left( {1 - {\upvarepsilon }} \right)\left( {{\uprho }_{{\text{p}}} - {\uprho }_{{\text{f}}} } \right){\text{g}}$$

where, ε is the bed porosity and is given as:

13 $$\varepsilon = 1 - \frac{{M_{bed} }}{{\rho_{p} V_{bed} }}$$

Substituting Eq. 13 in Eq. 12, and rearrangement gives:

14 $$M_{bed} = \frac{\Delta P}{{\Delta {\text{L}}}}\frac{{\rho_{p} V_{bed} }}{{\left( {{\uprho }_{{\text{p}}} - {\uprho }_{{\text{f}}} } \right){\text{g}}}}$$

At region ‘i’, the percentage mass of the bed can be calculated from the following equation:

15 $$\% M_{i} = \frac{{M_{i} }}{{M_{g} }}$$

Here ${M}_{i}$ is the mass of region ‘i’ and ${M}_{g}$ is the total bed mass; both values were calculated from the pressure drop using Eq. 14. The calculated region-wise mass percentage is reported in Table 2 for all the runs performed with zero primary flow. The mass of lower three regions are reported together since these bed regions were small as compared to middle $\left({\Delta P}_{4}\right)$ and upper $\left({\Delta P}_{5}\right)$ regions. It must be noted that ${\%M}_{i}$ values gives an insight of the bed during the steady conditions after the bed has completely expanded to the maximum extent and no further expansion is noticed. The mass has gradually increased in middle and upper regions with each run, which clearly indicates that the amount of fine particles has increased due to the deagglomeration in lower regions after every microjet flow run. Moreover, the particles’ size in lower region decreased in Run 2 but remained almost invariable thereafter in other runs. The phenomenon can be attributed to the initial turbulence, which subdued after Run 1, but the degree of deagglomeration in the impending runs was minimal in the lower region. Moreover, the powder which comprised of finer agglomerates rather than particulates in the middle region were deagglomerated in Runs 2–4, which in turn migrated in the upper layer, thus increasing the particulates in the uppermost region. The observed decrease in mass within the lower region directly corresponds to a reduction in agglomerate diameter, a relationship that can be established mathematically. First, according to Eq. (13), a decrease in the mass of solids within a given volume is direct consequence of an increase in the local bed voidage ($\varepsilon$). Second, the significant bed expansion from the microjet assistance indicates a particulate expansion regime, for which the Richardson-Zaki (R-Z) equation applies:

16 $$U_{r} = U_{t} \varepsilon^{n}$$

**Table 2:** Mass percentage at various bed regions for the different runs for zero primary flow runs.

	$Lower \, region$	$Middle \, region$	$Upper \, region$	$Bed \, mass \, error (\%)$
Run 1	42%	24%	32%	9.3%
Run 2	31%	28%	41%	4.0%
Run 3	32%	27%	43%	6.5%
Run 4	31%	22%	48%	2.0%

Here ${U}_{r}$ is the relative velocity between phases, ${U}_{t}$ is the terminal settling velocity of the agglomerates, and $n$ is the R-Z exponent. Since the gas velocity was constant throughout the experiment, the R-Z equation dictates that the observed increase in voidage ($\varepsilon$) must be accompanied by a decrease in the agglomerates’ terminal velocity (${U}_{t}$). Finally, as established by Stokes’ law (Eq. 17), a lower terminal velocity directly corresponds to a smaller agglomerate diameter (${d}_{p}$):

17 $$U_{t} = f\left( {d_{p}^{2} } \right)$$

To quantify the potential loss of nanosilica due to elutriation during the experiments, the bed mass error was calculated using the following equation:

18 $$Bed \, mass \, error \left( \% \right) = \left( {1 - \frac{Calculated \, mass}{{Measured \, mass}}} \right)$$

Here ‘measured mass’ refers is the initial mass of nanosilica added to the column. The ‘calculated mass’ is determined using Eq. 14 by replacing $\Delta P={P}_{g}$. The results, reported in Table 2, indicate that the mass error was small for most runs, which also helps verify the accuracy of the pressure drop measurements. However, a higher error of 9.3% was observed for the first run. This initial discrepancy is attributed to factors beyond elutriation, such as minor handling losses and the adhesion of particles to the column walls. These wall effects were significantly reduced after the initial fluidization, resulting in the much lower error values observed in all subsequent runs.

**Fig. 6:**
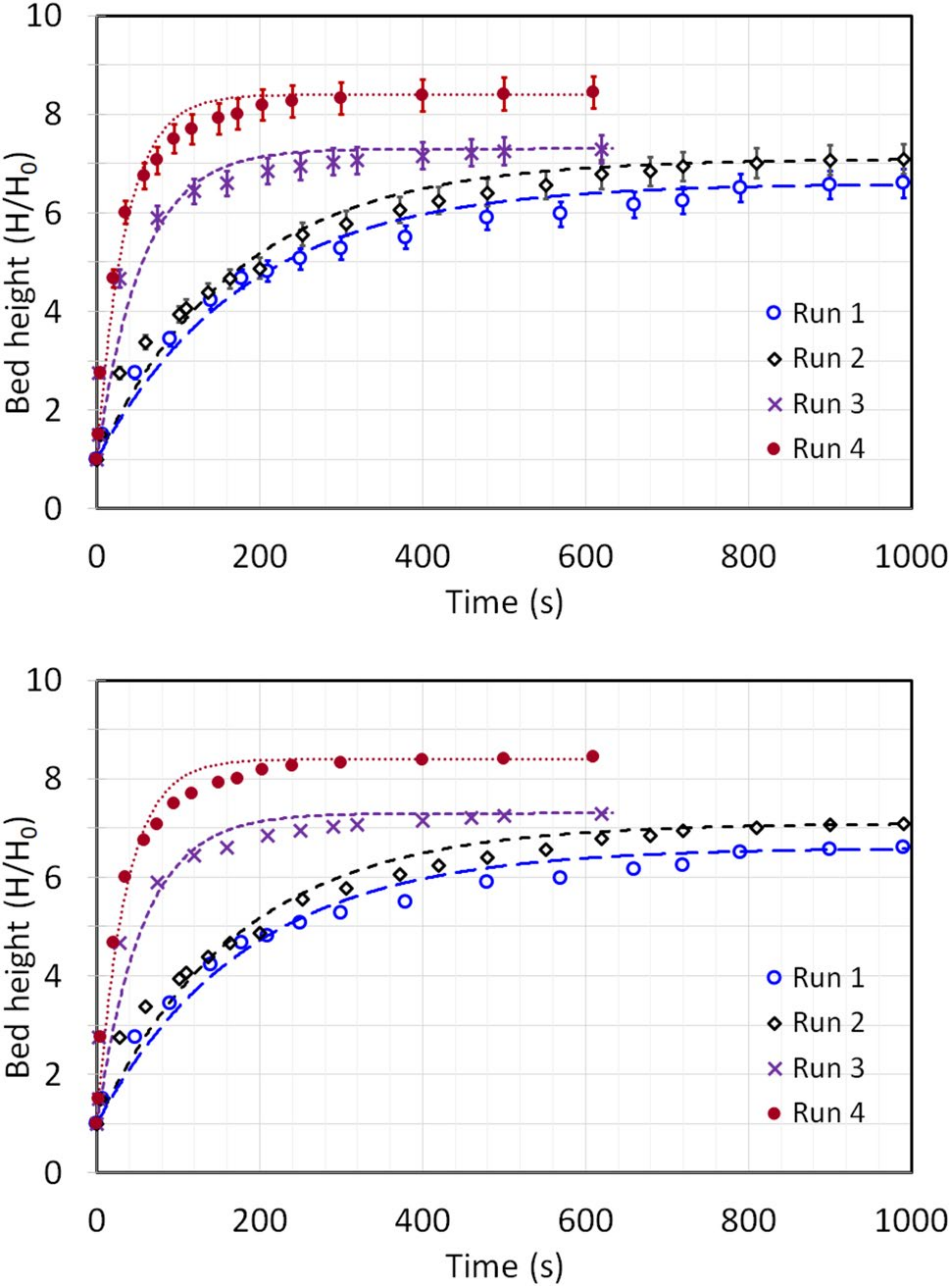
Change in bed height during the expansion with first order dynamics fit at zero primary flow and secondary flow of 312 m/s.

Figure 6 reports the change in height of the expanding bed for the consecutive runs, discussed in the preceeding paragraph. The maximum relative deviation of data during the repetition of experiments was found to be 4.3% indicated as error bars in the curves. The orientation of the curves closely resembles the dynamics of a first order system with a unit step change. The behavior of a first order system when a input step change is given to the system is characterized as:

19 $$y = K\left( {1 - e^{{{\raise0.7ex\hbox{${ - t}$} \!\mathord{\left/ {\vphantom {{ - t} \tau }}\right.\kern-0pt} \!\lower0.7ex\hbox{$\tau $}}}} } \right)$$

where ‘K’ is a constant refered to as gain and τ is the time constant.

The regression analysis of the bed height data with the above equation gives:

20 $$\frac{H}{{H_{0} }} = \left( {\left( {\frac{H}{{H_{0} }}} \right)_{max} - 1} \right)\left( {1 - e^{{{\raise0.7ex\hbox{${ - t}$} \!\mathord{\left/ {\vphantom {{ - t} \tau }}\right.\kern-0pt} \!\lower0.7ex\hbox{$\tau $}}}} } \right) + 1$$

The unit step change in the present process is the actuation of the microjet flow. The bed height ratio can be correlated with the bed voidage using Eq. 13 as:

21 $$\frac{H}{{H_{0} }} = \frac{{1 - \varepsilon_{0} }}{1 - \varepsilon }$$

The coefficient of determination (R^2^) values reported for Runs 1–4 in Table 3 signifies an optimal fit, the lowest R^2^ value is as high as 0.94.

**Table 3:** Bed height parameters calculated using Eq. 6 for zero primary flow runs.

	$${R}^{2}$$	$$\left( {\frac{H}{{H_{0} }}} \right)_{max} \left( {{\raise0.7ex\hbox{$ - $} \!\mathord{\left/ {\vphantom { - - }}\right.\kern-0pt} \!\lower0.7ex\hbox{$ - $}}} \right)$$	$$\tau (s)$$	T = $$5\tau (s)$$
Run 1	0.98	6.6	183.4	917
Run 2	0.97	7.1	173.6	868
Run 3	0.94	7.3	55.3	276.5
Run 4	0.98	8.4	35.1	175.5

The bed expansion was immense during the Run 1, which was as high as 6.6 times from the initial static bed height due to microjet effect (Table 3). For unassisted fluidation, the maximum bed expansion reported in the literature was around 1.4 times than the initial static bed^33^. In comparison with other techniques, the bed expanded 1.3 times for nanoparticles bed premixed with Geldart group A particles, while the bed expansion with pulsation assisted fluidization was around 1.7 times for hydrophillic nanopowder^37,51^. The Aerosil 200 nanosilica, which typilcally display ABF behavior due to their agglomeration tendency and size based segregation and poor bed expansion can successfully expand several folds under the asssitance of microjet fluidization^21,52^. The maximum bed height attained at steady state increased with the successive runs due to the increase in fine particles. The finer particles suspended higher in the column. The time constant of a process can be associated with the speed of response of a process; a lower value indicates a faster response. The response of the bed has improved in consecutive runs. The bed in Run 4 responded approximately 5.2 times quicker than Run 1 due to the fact that the bed was already homogeneous constituting already deagglomerated fine particles, which promptly expanded with the inception of microjet flow. Moreover, the τ values can also be used to calculate the time period of the stability of the process (steady state). From Eq. 20, at $t=5\tau$ the process output reaches at 99.3% to its maximum value. The stability time depicts similar behavior as discussed upon, further enumerating the above discussed justification.

### Microjet effect with varying primary flow and microjet flow of 312 m/s

**Fig. 7:**
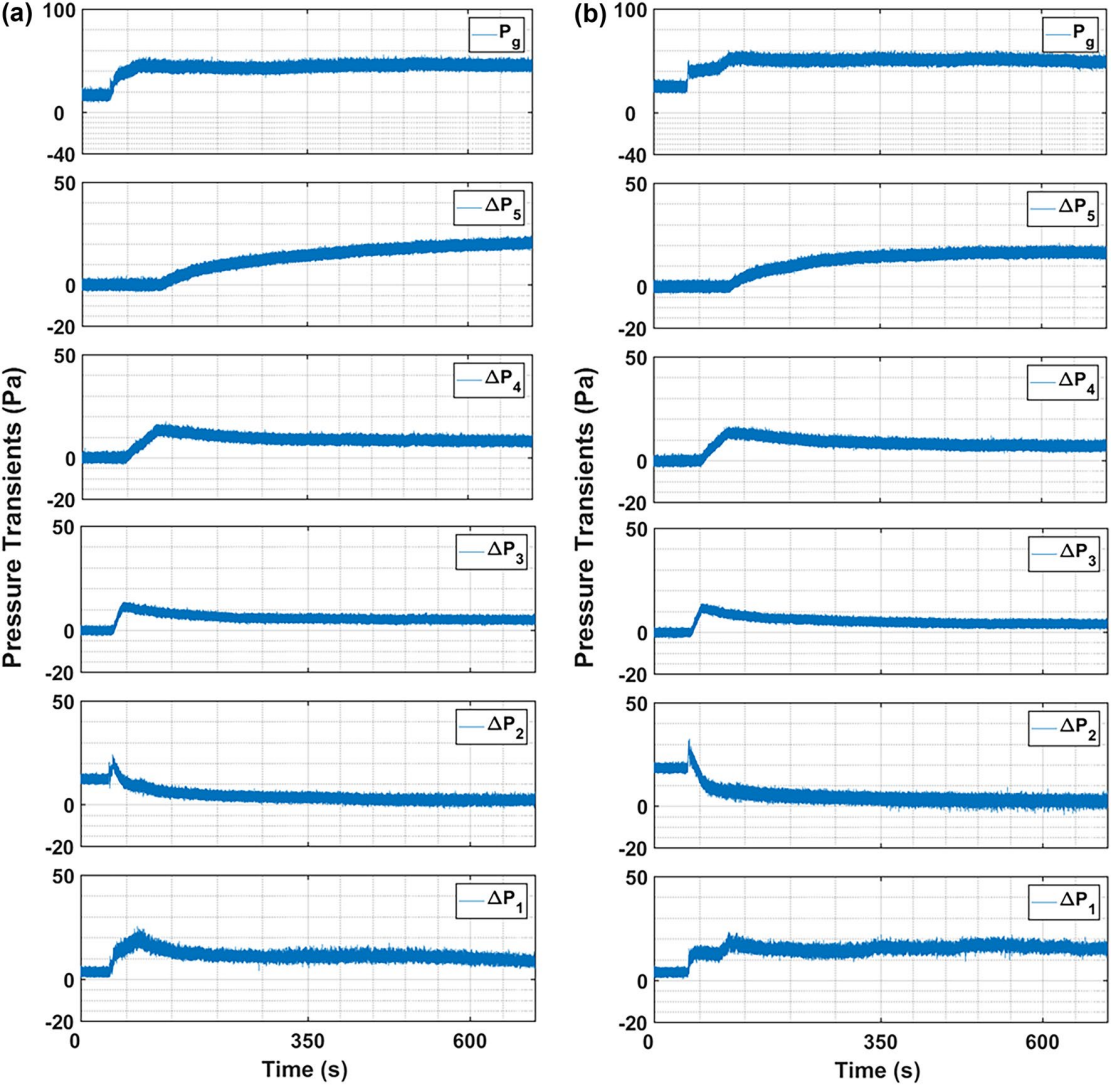

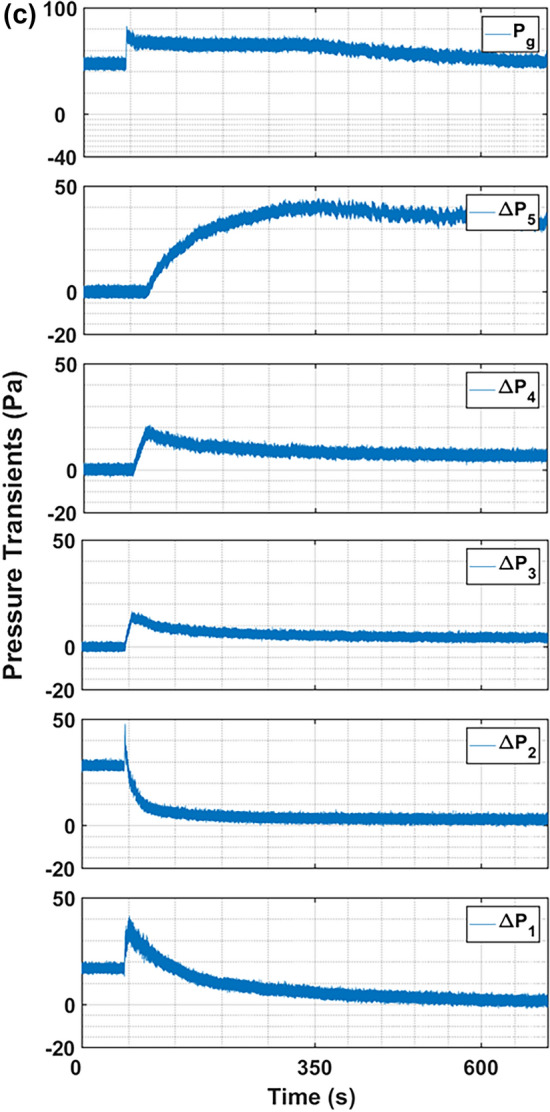
Region-wise pressure drop transients of nanosilica during the microjet flow with bed height 16 cm, when microjet flow is 312 m/s; (**a**) Primary flow = 0.5 U_mf_; (**b**) Primary flow = U_mf_; (**c**) Primary flow = 2 U_mf_.

**Fig. 8:**
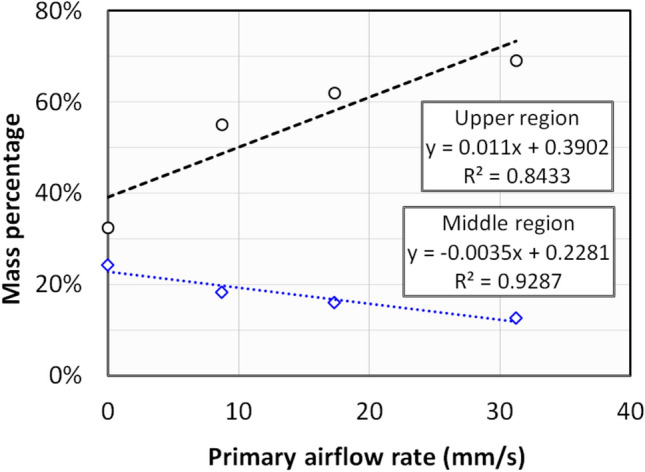
Mass concentration in upper region at different primary airflow rates with microjet assisted flow.

Figure 7 reports the pressure transients of the nanosilica bed at the primary flow of 9 mm/s ($0.5{U}_{mf}$), 17 mm/s (${U}_{mf}$) and 35 mm/s ($2{U}_{mf}$_)_ respectively^53^; and the secondary microjet flow was set at 312 m/s. Unlike the previous section, the nanosilica powder in the column was changed and the fresh nanosilica powder was used after every run here. The primary flow was initiated first and the bed was allowed to stabilize and thereafter the secondary microjet flow was introduced. In Fig. 7 a–c, the pressure drop value visible at ${\Delta P}_{1}$ and ${\Delta P}_{2}$ $\left(\Delta P\ne 0 Pa\right)$ during the initial data recording is due to the primary fluid flow in the column, which increased with the intensity of primary flow. The initial turbulence in ${\Delta P}_{1}$ region due to the microjet flow is feeble here as compared to the ‘zero primary flow’ run in Fig. 5– a. This could be due to the rearrangement of the bed due to the primary flow. The initial turbulence rather decreased at higher primary flow rates as seen in Fig. 7 b–c. In fact, for the case of $2{U}_{mf}$ primary flow in Fig. 7–c, the pressure drop decreased to around 1 Pa after the initiation of microjet from the initial pressure drop of 17 Pa. It must be noted that the bed was already in complete fluidized state at this fluid flow rate, and the additional energy by microjet resulted in further homogenization of the bed by deagglomeration resulting in more fine particles which shifted to the upper regions and higher bed expansion. Table 4 demonstrates the apparent impact of primary flow on the region-wise mass in the microjet assisted bed. The particles consistently decreased in lower region due to the higher deagglomeration phenomena. The particles’ mass decreased by 40% in the lower region until incipient fluidization. It must be noted that the bed behaviour was similar during packed bed and incipient fluidization. However, the particles’ mass decreased by around 60% at fully fluidized state compared to ‘zero primary flow’ run. The similar pattern was visible in middle region as well, the particles’ mass decreased consistently in the region with the increase in primary airflow rate. Besides, the amount of fine particles increased in upper region. The mass concentration of middle and upper regions is plotted in Fig. 8. The rate of increase in mass percentage in upper region is more than three times the decrease in middle region. This indicates that the fine particulates in the bed has increased with the primary airflow, which significantly aided the fluidized bed to attain high degree of bed expansion and deagglomeration.

**Table 4:** Mass percentage at various bed regions for the different runs for varying primary flow runs.

Primary fluid flow rate	$Lower \, region$	$Middle \, region$	$Upper \, region$
0 mm/s	42%	24%	32%
9 mm/s	24%	18%	56%
17 mm/s	25%	16%	61%
35 mm/s	16%	13%	69%

**Fig. 9:**
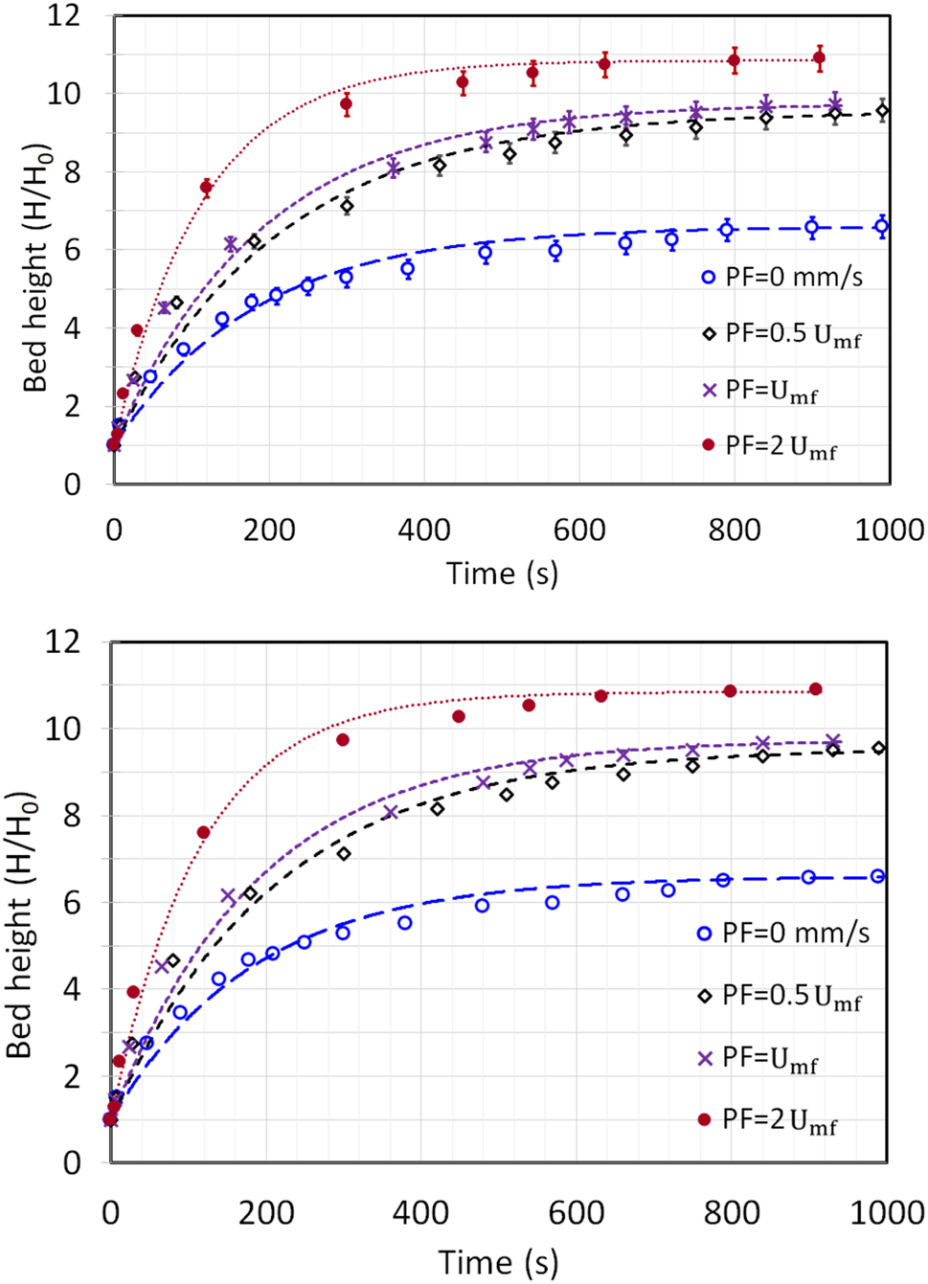
Change in bed height during the expansion with first order dynamics fit at varying primary flow and secondary flow of 312 m/s.

**Fig. 10:**
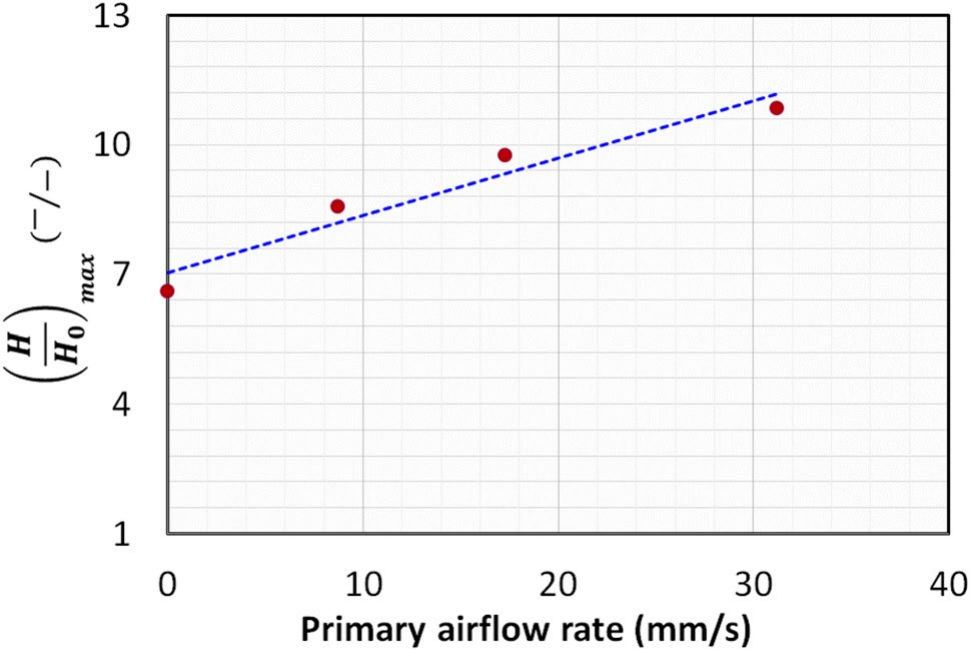
Change in maximum bed height attained at steady state at varying primary flow and secondary flow of 312 m/s.

The first-order system response model (Eq. 20) was fitted to the bed height data presented in Fig. 9. The resulting parameters such as maximum bed height attained and the time constant (τ) are reported in Table 5. The high R^2^ values signifies an excellent fit with minimal variation. The maximum relative deviation of data was 3.1% here. As shown in Fig. 10, ${\left(H/{H}_{0}\right)}_{max}$ increased linearly with the primary airflow rate. The bed expanded extensively, reaching approximately 10.9 times its static height (${\left(H/{H}_{0}\right)}_{max}=10.9)$ in the fully fluidized state at $2{U}_{mf}$. Analysis of the time constant (τ) reveals a more complex trend. At low primary airflow rates (packed bed regime), the system’s response time was surprisingly longer than that observed with no primary flow. This may be attributed to initial particle rearrangement increasing bed non-homogeneity. However, as the flow rate increased towards and beyond incipient fluidization, τ decreased markedly, indicating a quicker stabilization of the bed. The faster response, combined with the enhanced expansion, confirms that microjet-assisted fluidization aided by a primary airflow significantly improves fluidization quality, particularly by promoting a stable, expanded bed structure like agglomerate particulate fluidization (APF) especially after incipient fluidization is achieved.

**Table 5:** Bed height parameters calculated using Eq. 6 for varying primary flow runs.

Primary fluid flow rate	$$R^{2}$$	$$\left( {\frac{H}{{H_{0} }}} \right)_{max} \left( {{\raise0.7ex\hbox{$ - $} \!\mathord{\left/ {\vphantom { - - }}\right.\kern-0pt} \!\lower0.7ex\hbox{$ - $}}} \right)$$	$$\tau (s)$$	T = $$5\tau (s)$$
0 mm/s	0.98	6.6	183.4	917
9 mm/s	0.98	8.6	211.5	1058
17 mm/s	0.99	9.8	189.1	846
35 mm/s	0.99	10.9	113.3	567

## Conclusions

When the zero primary flow runs were repeated in the first set of experiments with the same nanosilica powder, a persistence effect was visible during the subsequent runs, whereby the particles did not agglomerate to their initial form after the bed was static and the bed retained the interparticle properties after microjet assistance. The τ values calculated from unit step input in first order system indicated that the bed response time improved and the bed attained steady state at lower time after every zero primary flow run. The bed expansion was extensive under the microjet assistance. The nanosilica bed expanded up to 6.6 times in the first run, while the bed expanded up to 8.4 times in the fourth repeated run. Moreover, the mass concentration in the upper region increased after every successive run and reached up to 48% of the total static mass in the fourth run with zero primary flow rate.

The primary flow further enhanced the expansion of the bed and improved the fluidization quality especially at incipient fluidization and thereafter at fluidized state. The bed expanded up to 8.6, 9.8 and 10.9 times respectively than the original static height when the primary flow was set at packed bed, incipient fluidization and fluidized bed. In addition, the mass concentration also increased with the increase in primary fluid flow rate; the mass concentration increased by more than the double in the upper region at fluidized state as compared to the zero primary flow run due to the increase of fine particulates in the region.

## Data Availability

The datasets generated during and/or analyzed during the current study are available from the corresponding author on reasonable request.

## List of symbols

$A$: Cross sectional area (m^2^)
$c$: Speed of sound (m/s)
$C_{p}$: Constant pressure heat capacity (J/mol/K)
$C_{v}$: Constant volume heat capacity (J/mol/K)
$$d_{p}$$: Particles diameter (m)
$g$: Acceleration due to gravity (m/s^2^)
$G$: Mass velocity (kg/m^2^/s)
$H$: Total bed height (m)
$H_{0}$: Initial height of the bed at time t = 0 (m)
$$K$$: Process gain
M: Mach number (-/-)
$M_{bed}$: Mass of particles in the bed (kg)
$M_{i}$: Mass of particles in region ‘i’ (kg)
$M_{g}$: Total mass of particles in the bed’ (kg)
$M_{w}$: Molecular weight (g/mol)
$n$: Richardson-Zaki exponent (-/-)
$\Delta P$: Pressure drop across ∆L (Pa)
$$p$$: Pressure (Pa)
$R$: Universal gas constant (J/mol/K)
$R^{2}$: Coefficient of determination (-/-)
$$T$$: Absolute temperature (K)
$$t$$: Time (s)
$U_{mf}$: Minimum fluidization velocity (mm/s)
$$U_{r}$$: Relative velocity (m/s)
$U_{t}$: Terminal settling velocity (m/s)
$$v$$: Fluid velocity (m/s)
$V$: Volumetric flow rate (LPM)
$V_{bed}$: Volume of particles in the bed (m^3^)
$w$: Mass flow rate (kg/s)
$y$: First order system output

## Greek letters

$$\varepsilon$$: Bed porosity (-/-)
$\varepsilon_{0}$: Initial bed porosity (-/-)
$\rho_{b}$: Bulk solid density (kg/m^3^)
$\rho_{f}$: Air density (kg/m^3^)
$\rho_{p}$: True solid density (kg/m^3^)
$\rho$: Air density (kg/m^3^)
$$\tau$$: Time constant (s)
